# Crime Scene Examination of a Suicidal Pact: A Case Report

**DOI:** 10.7759/cureus.31981

**Published:** 2022-11-28

**Authors:** Nilesh Devraj, Samarendra Barman, Vaibhav D Sonar

**Affiliations:** 1 Forensic Medicine, All India Institute of Medical Sciences, Guwahati, IND; 2 Forensic Medicine, Government Medical College and Hospital, Jalgaon, IND

**Keywords:** crime scene, post-mortem, forensic medicine, post-mortem examination, forensic medicine expert, crime scene examination, dyadic death, suicidal pact

## Abstract

A suicidal pact is a mutual agreement between two or more individuals to die together. In dyadic death, the assailant commits suicide soon after killing his victim. Cases of a suicidal pact and dyadic death are uncommon, especially in rural India. There are very few reported cases of suicidal pacts in the scientific literature. This present report highlights the death of three family members including a minor with the crime scene examination and challenges faced by the investigators and forensic experts. Being an expert in forensic medicine, we visited the crime scene and conducted post-mortem examinations to differentiate between suicidal pact and dyadic death.

## Introduction

A suicidal pact is defined as when two or more individuals commit suicide to end their life usually at the same time and place [1]. Dyadic death means an incident where homicide is committed and is followed by the perpetrator's suicide almost immediately or soon after the homicide [2,3]. Dyadic death involves the death of two or more people in one incident, usually involving family members or close relatives. The perpetrator is usually the elder person of the family or the one who is tremendously under stressful conditions and the victim usually involves children and females because of their non-supremacy in the family.

Usually, it is difficult for investigating agencies to differentiate between a suicide pact and dyadic death. Here, in this case, the investigating officer wanted to rule out the possibility of a suicide pact and dyadic death. They also wanted to rule out the involvement of the murderer behind the unnatural death of their fourth family member a few days back. Crime scene investigation is remarkably important to make a statement on the manner of death [4]. The crime scene visit, samples collected from the crime scene, and meticulous conduct of post-mortem examination by autopsy surgeon play a major role in making an evidence-based opinion on the manner of death and closure of such investigation. This type of medical examiner system of inquests is widely used in the United States, Canada, and Japan, and medical examiners are trained in both medicine (forensic pathology) and law. It is often regarded as the best inquest system in the world [5-7].

## Case presentation

**Figure 1:**
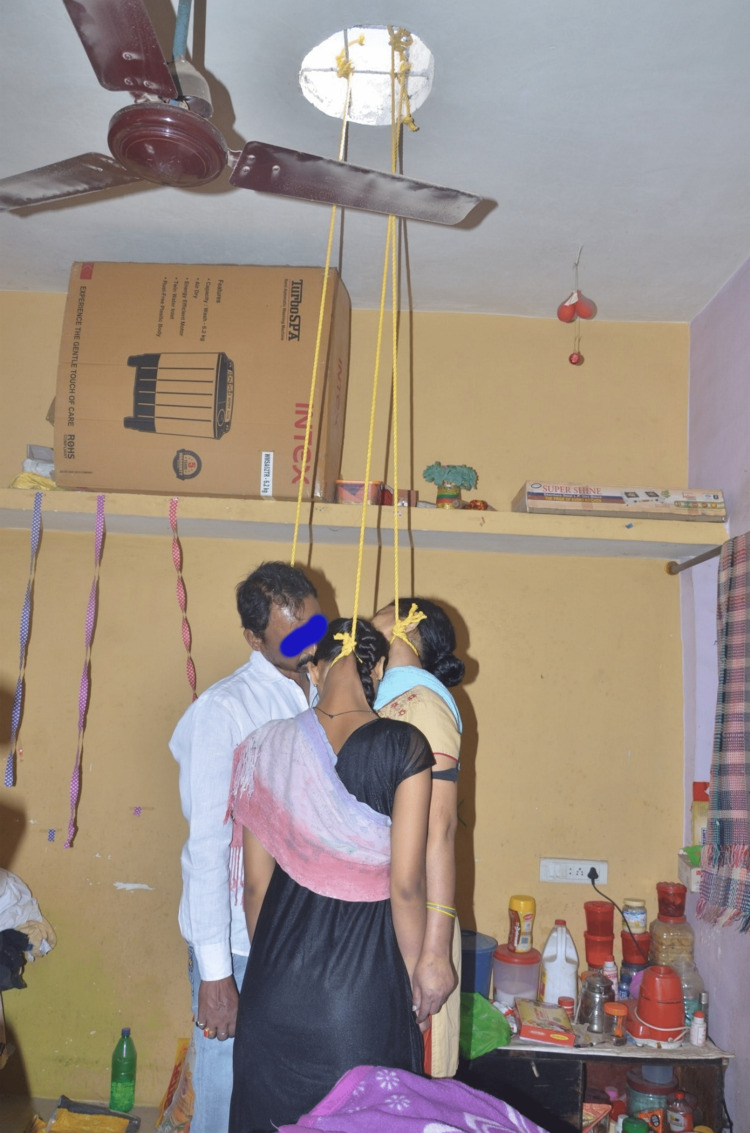
Three family members found in hanging condition

The lead author, while working as an assistant professor & professor and head of the department of forensic medicine at a state-run medical college located in Maharashtra was called on to provide his expert opinion regarding the deaths of three family members in a rural area located approximately 90 km away. The family members, including the husband (45 years old), wife (37 years old), and daughter (17 years old), were found hanging in their home without a sign of breach (Figure 1).

**Figure 2:**
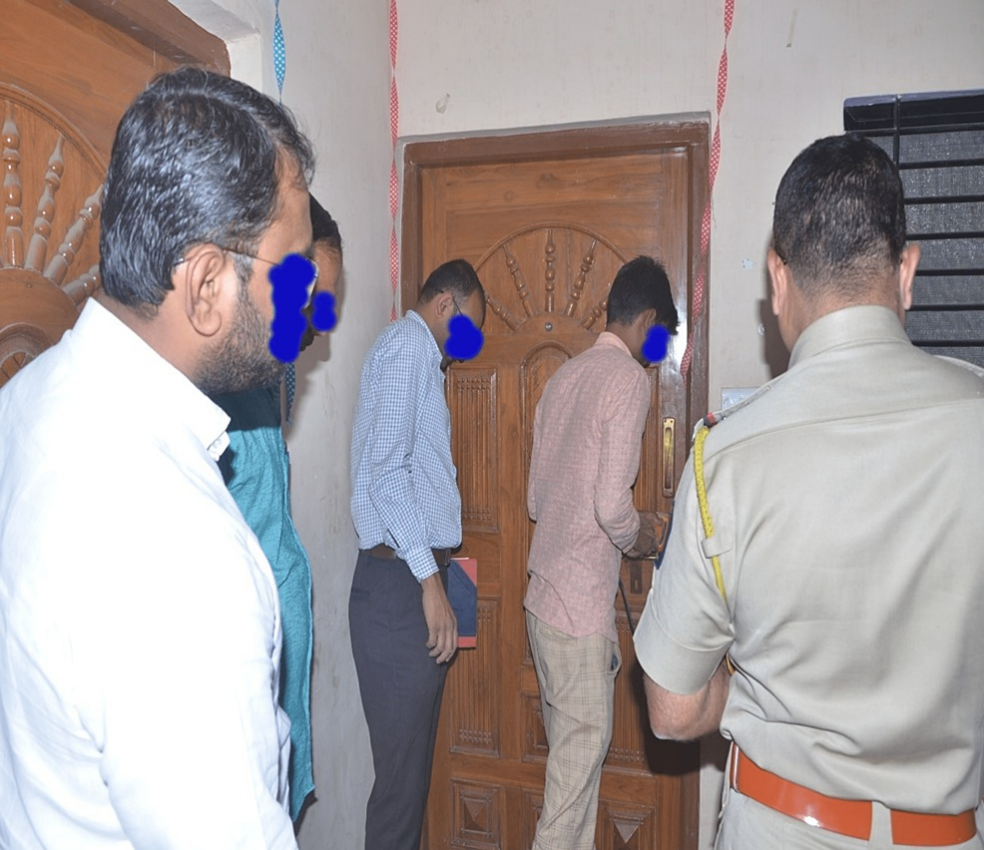
Police entering the house by breaking the latch of the main door

Their neighbors alerted local law enforcement authorities (state police) because family members were not responding to repeated calls. They also noted that no one came in or out of the house. Upon arrival, police officials had to break open the only door, which was locked from the inside (Figure 2).

The dead bodies were removed by police officials and transferred to a nearby rural hospital for post-mortem examination.

The investigating officer wanted to rule out the possibility of the involvement of the same person as the murderer behind the murder of the fourth member of the family and these three people, as a few days before, the fourth family member, a nine-year-old son, was killed. The son of the family was missing for the last seven days and was found in a naked, unconscious condition with multiple blunt injuries over the body in a forest area near the village. The law and order situation deteriorated as a result of both incidents, which occurred within one and a half weeks. The investigating officer had to keep all three bodies at the morgue of a rural hospital, as relatives demanded the post-mortem examination needs to be conducted by higher authorities from a state-run medical college. Thus, the investigating officer requested forensic medicine experts from the nearby government medical college to help them in the investigation. We, as a team of autopsy surgeons, first visited the crime scene before conducting the post-mortem examination.

**Figure 3:**
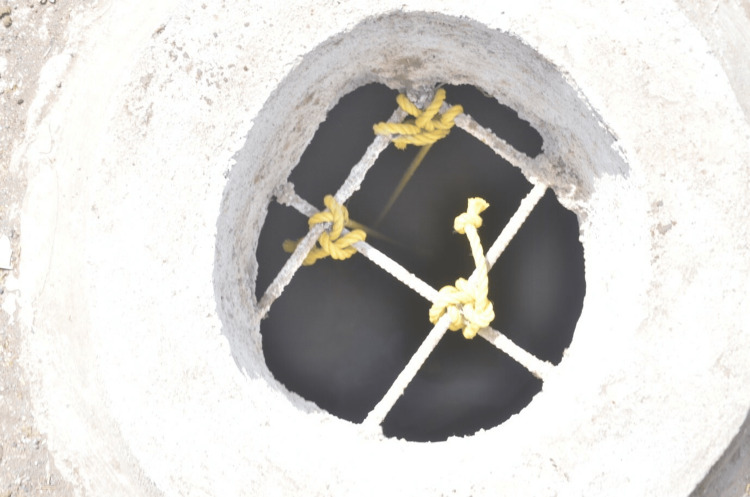
Roof having duct for ventilation along with three ligature material knots

The following observations were made during the crime scene examination and as per the spot inquest carried out by investigating officer. It was noted that the door had been breached by police to gain entry. There were no signs of any other breach. Household articles and effects appeared undisturbed indicating no signs of struggle. The house can be described as having two rooms with one being used as a hall and the other used as a kitchen cum bedroom. There was only one lavatory located outside the house. The bodies were found in hung condition in the kitchen cum bedroom. Nylon rope had been used to make three separate nooses, which were hinged from the ventilation duct in the roof of the kitchen cum bedroom (Figure 3).

**Figure 4:**
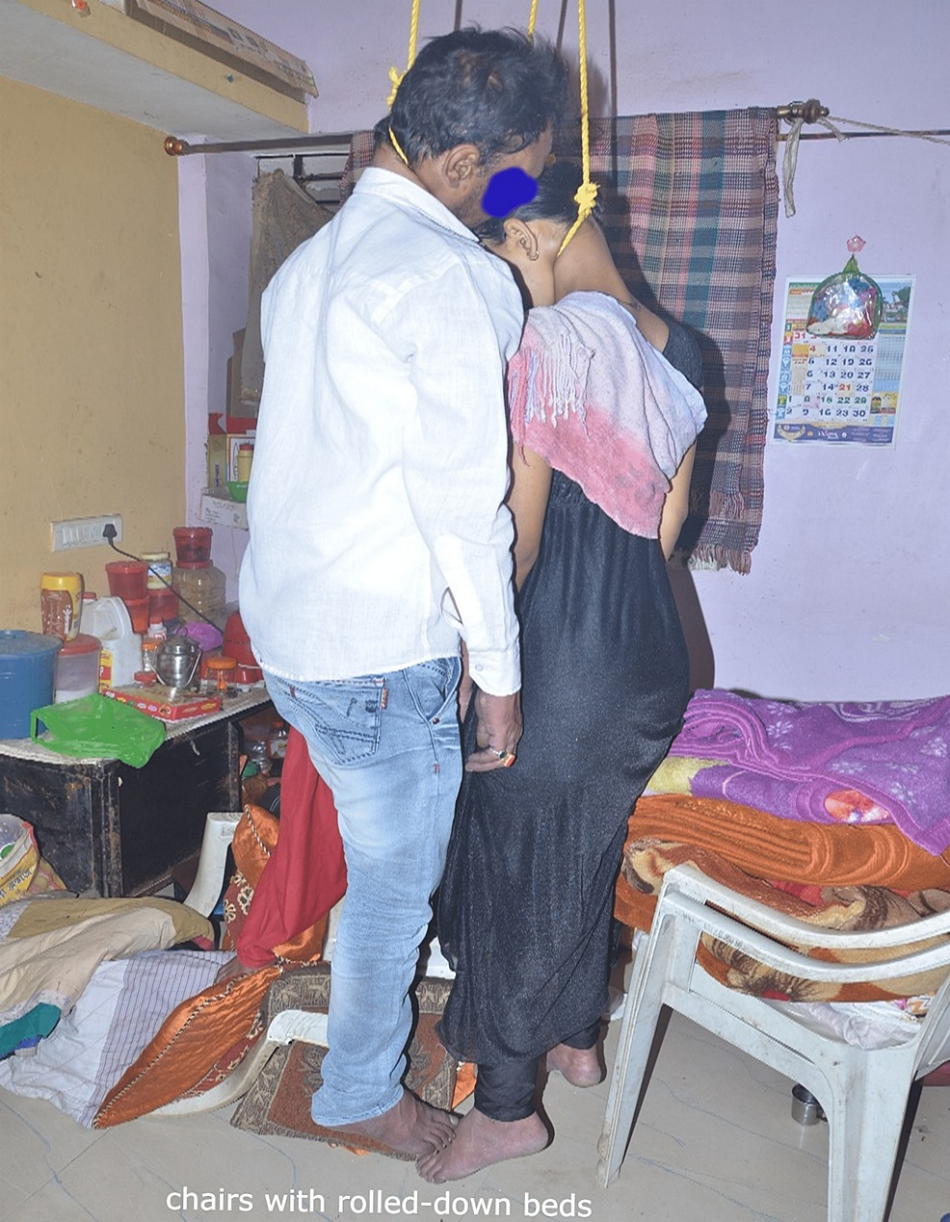
The deceased family members were found in hanging condition using chairs with rolled-down beds

The ventilation duct had a diameter of 20 cm, with multiple iron rods. The ligature material was already removed by the investigating officer before our crime scene visit. The photographs made available by the investigating officer showed the knot present over the roof appeared to be fixed. The ligature knot was present over the left lateral aspect of the neck of the husband (45 years old) and wife (37 years old) and just below the occipital protuberance of the head of the daughter (17 years old). The only knot visible among family members was over the daughter's head and appeared to be a running noose type. They used plastic chairs with rolled-down beds to climb (Figure 4).

We measured the height of the roof from the floor was 11.5 feet (350 cm). The length of the male, female, and daughter was 176 cm, 161 cm, and 143 cm, respectively. The height of the chair was 76 cm and the bed was 90 cm. The ligature material was already collected by police officials for chemical analysis while conducting a spot inquest before our visit. It was not possible to take the dimensions of the ligature material, as it was already collected and sealed by investigating officer to submit to the forensic science laboratory for chemical analysis.

After noting down our findings at the crime scene, we conducted a post-mortem examination of all three deceased at the morgue of the rural hospital with minimum available infrastructure. Based on the post-mortem examination findings of all three cases, our findings were directed toward asphyxia due to hanging and ante-mortem in nature. Finally, we gave our inference based on crime scene examination and post-mortem examination findings that this was a typical case of hanging and suicidal pact, where family members generally have a mutual agreement on a certain act and by which they perform this act without having a second thought.

It also came to light that the family was under tremendous stress and social stigma on account of an ongoing police investigation into the death of their son.

## Discussion

Any group of people such as romantic partners, couples, family members, or friends facing similar problems in life may enter into suicide pacts. Deaths following suicide pacts by family members may be referred to as "Family Suicide" [8,9]. These three family members died due to the hanging method and thus the cause of death may be identical in both deaths following a suicide pact and dyadic deaths. Hanging is the most commonly adopted method of dying by suicide in India. Death by hanging is also noted to be common in dyadic deaths [10-12]. However, the possibility of dyadic death was ruled out, as there were no signs of struggle. According to Locard’s exchange principle, there is an exchange of material evidence between the accused, victim, and the crime scene [13], but in this case, as per our observation, the crime scene was undisturbed, which rules out the involvement of any intruder and any signs of scuffle between family members. This shows their mutual agreement toward committing this action.

In India, the investigation into alleged suicides is conducted by investigating officers who are almost always police personnel with inadequate training in evidence gathering and crime scene forensics. Many times, details from the crime scene are missed, as these investigating officers do not visit the crime scenes. This approach can ensure that details from the crime scene are not overlooked, and cause and events leading up to death are conclusively established. This is also essential in differentiating deaths following suicide pacts and dyadic deaths. Aside from similarly advocating that crime scene visits are an essential part of the work of forensic medicine experts, Kumar et al. also insist that forensic medicine specialists be an integral part of investigating team [13]. Medical experts are involved in every aspect of death investigations and trials, including post-mortem examination, evidence gathering, physical examination, laboratory analysis, witness interviews, expert testimony, and depositions [14-17].

The current incident stands as a unique case as the investigating officer wanted to rule out the possibility of a dyadic death and involvement of the same person related to the murder of a nine-year-old son along with the other family members.

We observed several limitations during the investigation process such as poor training of the investigating officers to deal with such types of cases, inadequate mortuary-related infrastructure, and lack of forensic experts in the periphery hospitals.

## Conclusions

Crime scene examination helps investigators to reconstruct the event that leads to the death of the victim. Before conducting any post-mortem examination, it is always useful to conduct a crime scene investigation by a forensic expert to conclude the opinion and it is also useful for investigating agency too. Here, this case stands unique, in view of crime scene investigation carried out by a team of forensic experts prior to post-mortem examination. This helped not only them to give their opinion to differentiate between suicide pact and dyadic death but also helped the investigating officer to rule out the possibility of involvement of the lead family member in the murder of their son. The common intention of family members to perform this act related to their son’s murder drags them into tremendous stress and social stigma on account of an ongoing police investigation.
